# Moxibustion for rheumatoid arthritis: Protocol for a systematic review

**DOI:** 10.1097/MD.0000000000015899

**Published:** 2019-06-07

**Authors:** Xiao Wu, Yong Zhang, Bailu Chen, Jing Luo, Lu Gan, Guiquan Chen

**Affiliations:** Chinese Medicine Hospital Affiliated to Southwest Medical University, China.

**Keywords:** meta-analysis, moxibustion, protocol, RA

## Abstract

**Background:**

Rheumatoid Arthritis (RA) is a serious chronic disease which will result in serious syndrome such as joints stiffness, disability, and death. The major medications treating RA usually make sense and side effects, while moxibustion is known as a safe and effective treatment for RA. This review aims to systematically evaluate the effect and safety of moxibustion for treating RA.

**Methods:**

The following databases will be searched from their inception to March 2019: PubMed, Cochrane Central Register of Controlled Trials (CENTRAL), MEDLINE, EMBASE, Wan-Fang Databases, China National Knowledge Infrastructure (CNKI), Chinese Biomedical Literature Database (CBM), Citation Information by National Institute of Informatics, Chinese Scientific Journal Database (VIP Database). Two reviewers will search these databases, select data and measure the quality of studies independently. The methodological quality will be assessed by the Cochrane risk of bias tool. Data will be synthesized by either the fixed-effects or random-effects model according to a heterogeneity test. The primary outcome is symptom evaluation including morning stiffness, pain and joint swelling. The number of joints affected by RA, adverse effects, quality of life, erythrocyte sedimentation rate (ESR), C reactive protein (CRP), and Rheumatoid factor (RF) will be evaluated as secondary outcomes. Risk ratio for dichotomous data and mean differences with a 95% confidence interval for continuous data will be adopted to express the effect and safety of acupuncture for RA.

**Results:**

This study will provide a high-quality synthesis of current evidence of moxibustion for asthma from several aspects including morning stiffness, pain and joint swelling. The number of joints affected by RA, adverse effects, quality of life, erythrocyte sedimentation rate (ESR), C reactive protein (CRP), and Rheumatoid factor (RF).

**Conclusion:**

The conclusion of our study will provide updated evidence to judge whether moxibustion is an effective and safe intervention for patients with RA.

**Ethics and dissemination:**

As individuals will not be involved, the ethical approval will not be required. This review will be published in a peer-reviewed journal or at a relevant conference.

**Prospero registration number:**

CRD42019126685.

**Key Points**

This review will provide a systematic, objective, and comprehensive assessment of the effectiveness and safety of moxibustion for patients with rheumatoid arthritis.
This review will provide new and useful information for doctors, patients, and policymakers.
The literature searching, trial screening and data extraction will be conducted independently by 2 authors.
The languages only involved Chinese and English during electronic search will ignore the articles in Japanese, Korean, or other words.
The various kinds of moxibustion and different stages may increase the risk of heterogeneity.

## 1 Introduction

Rheumatoid arthritis (RA), one of the most prevalent chronic inflammatory diseases, is well-characterized by a circadian rhythm of clinical manifestation more serious in the early morning, while relieved to a relative stable condition in the afternoon.^[1–3]^ As a chronic polyarthritis of unknown etiology, RA has an incidence of 0.5% to 1%,^[4,5]^ and may result in joint destruction, disability, death, and so on.^[6–8]^ Currently, modern medical treatments for RA mainly focuses on symptomatic treatments such as anti-inflammatory, anti-rheumatic, and analgesic. However, increasing number of side effects cannot be ignored.^[9]^ Meanwhile, according to significant effect and safety, acupuncture and moxibustion for treating RA are becoming more and more popular.^[10–14]^ Previous systematic reviews have displayed acupuncture is effective and safe and may have some advantages over routine medical treatment.^[15–17]^ In addition, moxibustion is able to relieve RA in various pass ways, and there are lots of verifying study results to confirm the effects and safety of moxibustion for RA.^[18]^ However, there is still no critically designed systematic review to assess the effectiveness and safety of moxibustion for RA. Therefore, we will perform a systematic review of moxibustion for RA to collect some reliable evidence for clinical guidance and to assistant RA patients to seek more reasonable treatments. In this review, we aim to conduct a systematic review to evaluate all the clinical studies on the effects and safety of moxibustion for treating RA in adults.

## 2 Objective

The objective of this review is to systematically evaluate the effect and safety of moxibustion for treating RA.

## 3 Methods

### 3.1 Study registration

This protocol for the systematic review has been registered on PROSPERO (registration number: CRD42019126685). The review reporting will be conducted in compliance with the preferred reporting items for systematic reviews and meta-analyses (PRISMA) statement guidelines.

### 3.2 Study design

#### 3.2.1 Type of studies

All prospective randomized controlled clinical trials (RCTs) and quasi-RCTs will be included.

### 3.3 Type of participants

Patients suffered from RA will be included without sex, age, course, ethnicity, disease duration or disease severity restrictions.

### 3.4 Type of interventions

Any type of moxibustion therapy such as direct moxibustion, indirect moxibustion, heat-sensitive moxibustion, warm needling, natural moxibustion, moxa-burner moxibustion, crude drug moxibustion as the sole treatment or a part of combination therapy with other intervention will be included. Studies in which moxibustion is not used as a major intervention will be excluded.

### 3.5 Type of outcome measures

#### 3.5.1 Primary outcomes

Symptoms assessment including morning stiffness, pain, and joint swelling.

(1) The number of swelling joints affected by RA.
(2) The number of painful joints affected by RA.
(3) The duration of morning stiffness.

### 3.6 Secondary outcomes

(1) Pain visual analog scale (VAS) score.
(2) Physician VAS score.
(3) Adverse effects.
(4) Quality of life.
(5) Erythrocyte sedimentation rate.
(6) C reactive protein.
(7) Rheumatoid factor.

### 3.7 Search methods

#### 3.7.1 Searching databases

##### 3.7.1.1 Electronic searches

A literature search will be conducted by BC, JL, and LG in the databases of PubMed, Cochrane Central Register of Controlled Trials, MEDLINE, EMBASE, Wan-Fang Databases, China National Knowledge Infrastructure, Chinese Biomedical Literature Database, Citation Information by National Institute of Informatics, Chinese Scientific Journal Database (VIP Database) from their inception to March 2019.

### 3.8 Searching other sources

PROSPERO, the International Clinical Trials Registry Platform, and Clinical Trials.gov will also be searched to identify systematic reviews or ongoing/completed clinical trials. In addition, other resources will be searched manually such as the references of all included studies of this systematic review and general review. Theses and bibliographic references of included trials will also be reviewed.

### 3.9 Search strategy

The search strategy for electronic databases will adopt following items. Terms to be used for searching include (moxibustion OR moxa OR warm needling OR vesiculation OR blister) AND (rheumatoid arthritis OR RA OR RhA OR juvenile rheumatoid arthritis OR [nodule AND arthritis] OR joint stiffness OR morning stiffness OR joint pain OR joint swelling OR [joint stiffness AND joint pain AND joint swelling]).

Specific search strategies (eg, for PubMed) are as follows:

#1. ((((moxibustion [Title/Abstract]) OR moxa [Title/Abstract]) OR warm needling [Title/Abstract]) OR vesiculation[Title/Abstract]) OR blister [Title/Abstract].

#2. ((((((rheumatoid arthritis [Title/Abstract]) OR RA [Title/Abstract]) OR RhA [Title/Abstract]) OR juvenile rheumatoid arthritis [Title/Abstract]) OR nodule arthritis [Title/Abstract]) OR joint stiffness [Title/Abstract]) OR morning stiffness [Title/Abstract]) OR joint pain [Title/Abstract]) OR joint swelling [Title/Abstract].

#3. (((random [Text Word] OR randomized [Text Word]) OR control [Text Word]) OR controlled [Text Word]) OR trial [Text Word] AND “humans” [MeSH Terms]

#4. #1AND#2AND#3.

We will use similar search strategies for other electronic databases.

### 3.10 Study selection

**Figure 1:**
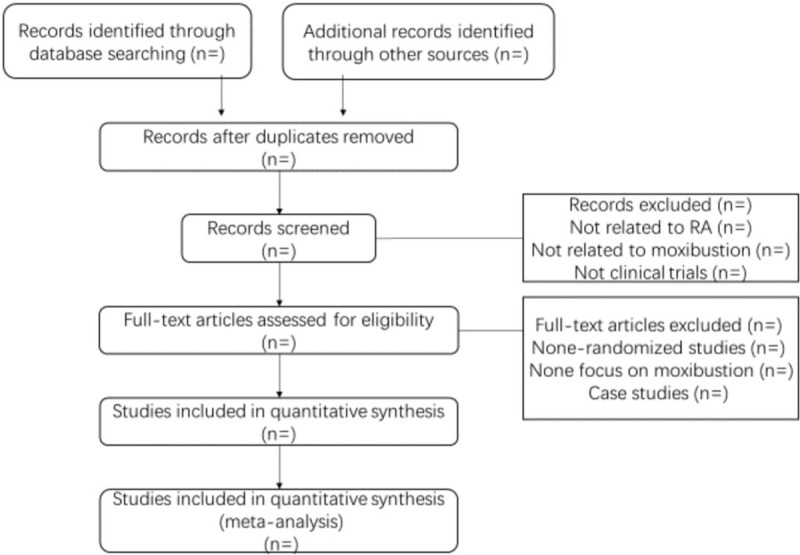
The PRISMA flow chart. PRISMA = preferred reporting items for systematic review and meta-analysis.

The data screening and selection process will be performed independently by BC and LG, then will be verified by XW. When disagreements on the selection are not resolved through team discussion, XW will make the decision. The details of selecting process will be presented in the PRISMA flow chart (see Fig. 1).

### 3.11 Data extraction

Before beginning extraction, a consistency assessment will be performed through a pilot test, in which each of them will evaluate 2 trials, respectively. After making a common consensus, we will use a predefined extraction form to collect data from included trials, involving general information (name and year of publication, date of extraction, title of study, and author's publication details), study characteristics, eligibility criteria, interventions, outcome measurements, duration, adverse events, results, and type of moxibustion. For literature published in abstract or dissertation form with important details regarding methods and results missing, we will try to retrieve information from the authors.

### 3.12 Quality assessment

The Cochrane risk of bias tool, which is recommended by the Cochrane Reviewer's Handbook 5.0.24, will be used to evaluate the quality of the included studies. BC and LG will independently evaluate the quality of selected articles from the following 5 aspects: selection bias (random sequence generation or allocation concealment), performance bias and detection bias (blinding), attrition bias (incomplete outcome data), reporting bias (selective outcome reporting), and other biases. If necessary, we will contact the corresponding author to clarify issues. The result of the consistency evaluation will be presented with Kappa statistics, Kappa value <0.75 will be considered the consistency have reached. Any disagreements will be resolved through discussion or consultation with XW.

### 3.13 Data synthesis

Depending on its characteristics (participants, interventions, comparisons, and outcomes or methodology) of collected data, Analysis will be different. If there is excessive data heterogeneity, qualitative analysis will be performed to summarize the study. When meta-analysis is feasible, quantitative analysis will be used to process data, and each type of moxibustion intervention will be evaluated separately.

The review manager (RevMan V.5.3) software for Windows will be employed to carry out all statistical analyses. Before data meta-analysis, heterogeneity will be tested with a standard *χ*^2^ test. For studies with high heterogeneity (*P* > .10, *I*^2^ ≤ 50%), the fixed-effect model will be employed. For dichotomous and continuous data, the relative risk and mean difference with 95% confidence intervals will be expressed for evaluations respectively. For studies with low heterogeneity (*P* ≤ .10), subgroup or sensitivity analysis will be conducted. Ultimately, the total effect will be measured by using the *Z* score with significance set at *P* ≤ .05. Funnel plots will be used to detect the possibility of publication bias.

### 3.14 Subgroup analysis

If the necessary data are available, subgroup analysis will be conducted according to different factors as follows:

(1) Control interventions, such as sham/placebo moxibustion, no treatment, other traditional Chinese medicine (TCM) treatment or non-TCM treatment.
(2) Type of moxibustion, such as direct moxibustion, indirect moxibustion, beat-sensitive moxibustion, moxa burner moxibustion, warm needling, crude drug moxibustion or natural moxibustion.
(3) Treatment frequency, such as less than 3 times per week versus more than 3 times per week.
(4) Duration of RA.
(5) Laterality of RA, bilateral RA versus unilateral RA.

### 3.15 Sensitive analysis

Sensitive analysis will be performed after excluding low-quality articles to identify whether the conclusions are robust. The “risk of bias” tool will be used to evaluate the methodological quality of studies. The lower quality article that has more than 3 “risk of bias categories” graded will be excluded, then we will carry out a second meta-analysis. The results of the 2 meta-analyses will be compared and discussed.

### 3.16 Patient and public involvement

Even though the patients will not be involved in the design of this study, development of the research question and outcome measures will be informed by patients’ priorities, experience and preference as reported in the published clinical studies in this domain. The results of this review will provide patients with new information on the credibility of current non-pharmacological treatments for treating RA.

## 4 Discussion

RA is a chronic systemic autoimmune disease, which can cause cartilage and bone damage as well as disability, and carries a substantial burden for both the individual and society.^[19]^ Studies have reported that moxibustion could relieve the RA related syndromes such as joints stiffness, joint pain and to prevent joint destruction and disability.^[14,20–22]^ However, the quality of current evidence has not been determined. Consequently, we will carry out a systematic review to provide more verifying evidence for doctors and patients. And the systematic review will provide a detailed summary of the present evidence for the effects of moxibustion in treating RA.

While there may be some potential limitations of the review. For example, the languages only involved Chinese and English during electronic search will ignore the articles in Japanese, Korean or other words. Otherwise, the various kinds of moxibustion and different stages may increase the risk of heterogeneity. Finally, difficulty in blinding measures during moxibustion may lead to bias.

## Author contributions

GC is the guarantor of the article. XW designed and wrote the protocol, YZ advised on the design and performed data collection and analysis, BC, JL, and LG searched the literature, selected studies, and assessed data quality. All authors read and approved the final manuscript.

**Data curation:** Yong Zhang, Bailu Chen, Jing Luo, Lu Gan.

**Formal analysis:** Yong Zhang, Bailu Chen, Jing Luo.

**Methodology:** Xiao Wu, Guiquan Chen.

**Project administration:** Xiao Wu, Guiquan Chen.

**Resources:** Bailu Chen, Jing Luo, Lu Gan.

**Supervision:** Xiao Wu, Guiquan Chen.

**Validation:** Yong Zhang, Guiquan Chen.

**Visualization:** Yong Zhang.

**Writing – original draft:** Xiao Wu.

**Writing – review and editing:** Xiao Wu.
